# Supplementary figures and images for: Human Taste Cells Express ACE2: a Portal for SARS-CoV-2 Infection
Source: bioRxiv. 2021 Apr 21:2021.04.21.440680. Preprint. [Version 1] doi: 10.1101/2021.04.21.440680 (PMC8077572; doi:10.1101/2021.04.21.440680)

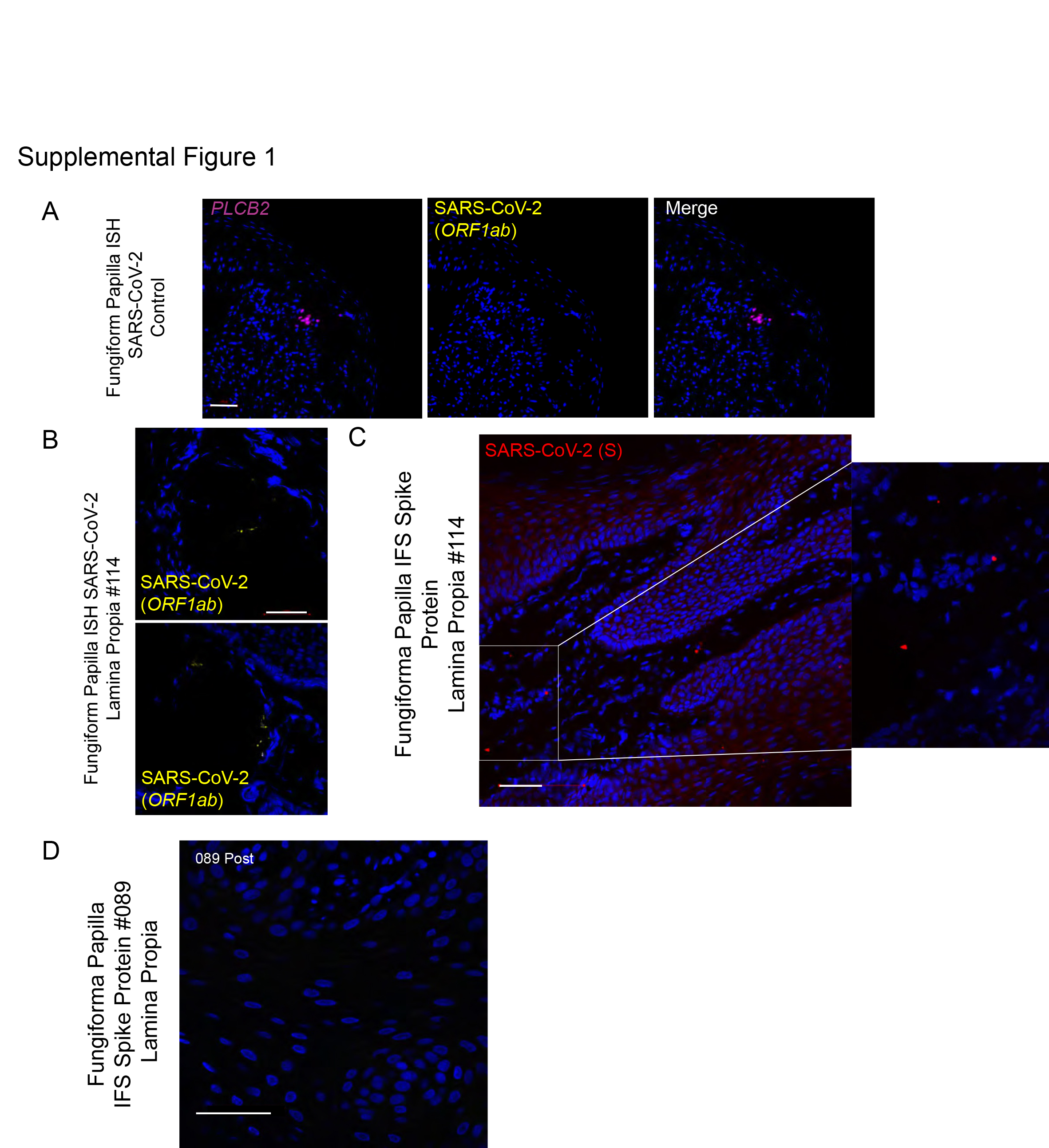

Supplement: Supplement 1 — Supplemental Figure 1. Panel A shows RNAscope detection in a taste bud of a fungiform papilla (FP) of the transcript for the type II cell marker PLCB2 and is the negative non-infected control for the SARS-CoV-2 ORF1ab probe in a taste bud biopsied from a non-infected age and sex matched control for #114. Nuclei stained by DAPI, shown in blue. Panel B shows the sense probe targeting the viral ORF1ab mRNA demonstrates positivity within the lamina propria of participant #114 during infection. Panel C shows immunostaining in red for the SARS spike protein in the lamina propria of a FP from participant #114 during infection. Panel D there is no evidence of SARS spike protein in the lamina propria of participant #089 post-COVID-19. Scale bars = 50μm. [file media-1.jpg]
